# Supplementary material for: Inhibitory Mechanism of Advanced Glycation End-Product Formation by Avenanthramides Derived from Oats through Scavenging the Intermediates
Source: Foods. 2022 Jun 20;11(12):1813. doi: 10.3390/foods11121813 (PMC9222356; doi:10.3390/foods11121813)
Supplement: Supplementary file 1 [file foods-11-01813-s001.zip › foods-1718353-supplementary.pdf]

Supporting Information

Inhibitory mechanism of advanced glycation end-product formation by avenanthramides derived from oats through scavenging the intermediates

Pei Zhu, Ying Zhang, Dianwei Zhang, Luxuan Han, Huilin Liu*, and Baoguo Sun

Beijing Technology and Business University, 11 Fucheng Road, Beijing, 100048, China.

***Corresponding author: Huilin Liu**

Tel: (86 10) 68984003;

Fax: (86 10) 68984857;

Email: liuhuilin@btbu.edu.cn

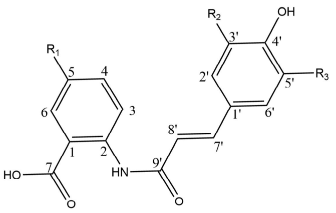

AVAs	R_1	R_2	R_3
2c	OH	OH	H
2f	OH	OCH_3	H
2p	OH	H	H

Figure S1 The chemical structure of AVAs 2p, AVAs 2c and AVAs 2f.

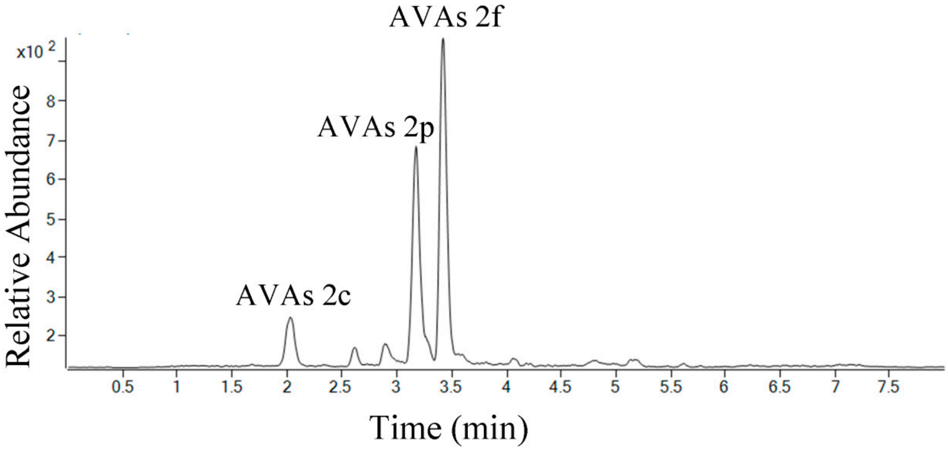

Figure S2 HPLC chromatogram of AVAs extracts.

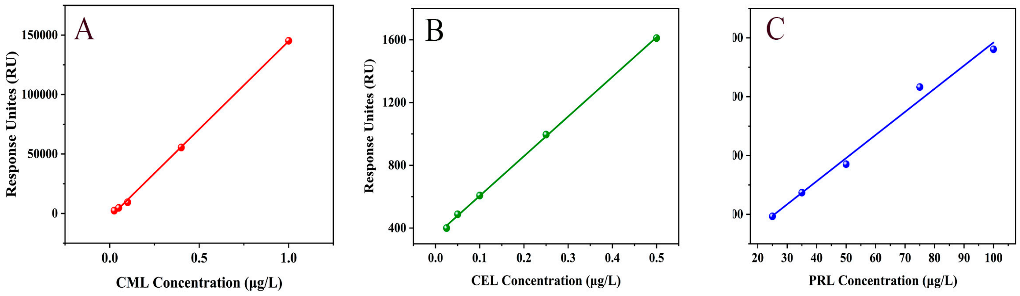

Figure S3 The standard curve of CML, CEL and PRL.

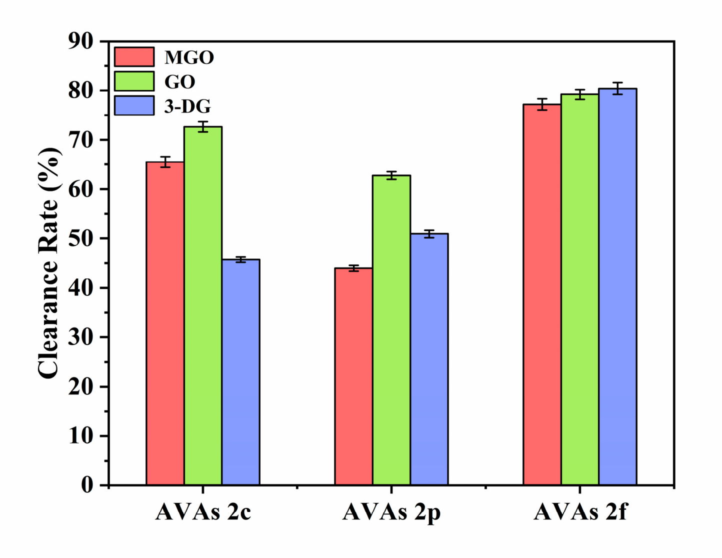

Figure S4 The clearance of the dicarbonyl compounds MGO, GO and 3-DG by AVAs 2p, 2c and 2f.
